# Crystal Structure of the HIV-2 Neutralizing Fab Fragment 7C8 with High Specificity to the V3 Region of gp125

**DOI:** 10.1371/journal.pone.0018767

**Published:** 2011-04-26

**Authors:** Hannes Uchtenhagen, Rosmarie Friemann, Grzegorz Raszewski, Anna-Lena Spetz, Lennart Nilsson, Adnane Achour

**Affiliations:** 1 F59 Department of Medicine, Center for Infectious Medicine (CIM), Karolinska University Hospital Huddinge, Karolinska Institutet, Stockholm, Sweden; 2 Department of Cell and Molecular Biology, Molecular Biophysics, Biomedical Center, Uppsala University, Uppsala, Sweden; 3 Department of Biosciences and Nutrition, Center for Biosciences, Karolinska Institutet, Huddinge, Sweden; Monash University, Australia

## Abstract

7C8 is a mouse monoclonal antibody specific for the third hypervariable region (V3) of the human immunodeficiency virus type 2 (HIV-2)-associated protein gp125. The three-dimensional crystal structure of the Fab fragment of 7C8, determined to 2.7 Å resolution, reveals a deep and narrow antigen-binding cleft with architecture appropriate for an elongated epitope. The highly hydrophobic cleft is bordered on one side by the negatively charged second complementarity determining region (CDR2) and the unusually long positively charged CDR3 of the heavy chain and, on the other side, by the CDR1 of the light chain. Analysis of 7C8 in complex with molecular models of monomeric and trimeric gp125 highlights the importance of a conserved stretch of residues FHSQ that is localized centrally on the V3 region of gp125. Furthermore, modeling also indicates that the Fab fragment neutralizes the virus by sterically impairing subsequent engagement of the gp125 trimer with the co-receptor on the target cell.

## Introduction

The human immunodeficiency virus 2 (HIV-2) was first described in the mid 1980s. It is closely related to HIV-1 and has a very similar genomic organization [1]. Nevertheless, the sequence identity of the two viruses is limited and HIV-2 is less pathogenic in humans. The transmission frequency of HIV-2 is reduced and infection results in lower viral load and longer latency with markedly slower progression to AIDS when compared to HIV-1 [2], [3]. Importantly, and in contrast to HIV-1, HIV-2 was also found to be relatively more susceptible to antibody neutralization [2].

The HIV-2 envelope proteins gp36 and gp125 are the main targets for neutralizing antibodies [4]. Although the crystal structures of gp36 and gp125 have not been determined yet, functional and structural studies suggest strong similarities to their HIV-1 homologues gp41 and gp120, respectively [2], [5]. As in HIV-1, they mediate viral fusion via binding to CD4 and a co-receptor, mainly CCR5 or CXCR4, on the target cell. The HIV envelope proteins assemble into surface spikes composed of trimers of non-covalent gp36-gp125 heterodimers, with gp36 traversing the viral membrane and anchoring the gp36-gp125 complex to the virus [6], [7]. The sequence of the heavily glycosylated gp125 protein is highly variable, especially within its five variable regions designated V1 to V5 that are considered crucial elements of neutralization resistance [8], [9].

The third variable region, V3, of gp125 consists of 35 amino acid residues presumably forming an exposed and flexible region linked at its base by a disulfide bridge between the cysteine residues C311 and C344 (according to the SIV Mac239 enumeration, http://www.hiv.lanl.gov). The V3 region is of crucial importance for co-receptor binding and determines, at least partially, the tropism of the virus [10], [11], [12]. Furthermore, infected individuals and animals frequently display high titers of neutralizing antibodies against V3. Accordingly, the V3 regions of gp120 and gp125 have been described as principal neutralization determinants for antibody responses against both viruses [13], [14], [15]. However, especially in HIV-1, this region is assumed to be partially masked prior to CD4 engagement and prone to escape from neutralization [10]. Conversely, the V3 region of gp125 in HIV-2 was found to be generally less variable and more accessible, which might contribute to its reduced neutralization resistance [10], [16].

For HIV-1, information derived from structural studies of Fab fragments of V3-specific antibodies, combined with the determination of the crystal structure of the V3-containing gp120 core, have contributed to a better understanding of epitope binding and of the structural basis for neutralization breadth [17], [18], [19], [20], [21], [22], [23]. In contrast, no similar structural information has been hitherto gathered for HIV-2, especially in the light of its disparate V3 sequence, lower resistance to neutralization and tendency for CD4 independence.

Two highly conserved immunodominant motifs have been previously described in the V3 region of gp125, corresponding to the stretches of residues 330–333 (FHSQ) and 343–345 (WCR) [24], [25], [26], [27]. FHSQ- and WCR-specific HIV-2 neutralizing V3-specific murine monoclonal antibodies have been isolated from animals immunized with V3-derived peptides [26], [27], [28]. The monoclonal antibody 7C8 binds to V3-peptides corresponding to the stretch of residues 326 to 341 that contains the FHSQ epitope [27], [29], [30]. Interestingly, the third complementarity determining region of the heavy chain (CDRH3) of 7C8 comprises 13 amino acid residues while the majority of CDRH3 domains from murine IgG_1_ are only 8 to 9 residues long [30], [31], [32]. This concords well with data from human neutralizing antibodies specific for gp120 and suggests an important role for the elongated CDRH3 loops.

In this study the crystal structure of the Fab fragment of 7C8 is presented, providing the first structural analysis of an HIV-2-neutralizing antibody. The three-dimensional structure of 7C8 reveals a deep and narrow highly hydrophobic antigen-binding site, bordered by the unusually long CDRH3 and the two CDRH2 and CDRL1 loops. A potential mechanism for viral neutralization through sterical hindrance by the 7C8 Fab fragment is proposed based on molecular modeling of the complex of 7C8 with the gp125 trimer.

## Results and Discussion

### Determination of the crystal structure of the Fab fragment of 7C8

The three dimensional structure of the Fab fragment of the HIV-2-neutralizing V3-specific monoclonal antibody 7C8 was determined to a resolution of 2.7 Å (Table 1) with R_cryst_ and R_free_ factor values of 22.6% and 27.3%, respectively. The asymmetric unit includes two 7C8 Fab molecules, 78 water molecules and one glycerol molecule. The model displays good stereochemistry with the exception of the valine residue V51 found in the disallowed region of the Ramachandran plot in both 7C8 monomers. Valine V51 is the *i*+1 residue of a distorted turn forming the CDRL2 loop and its deviation from ideal geometry is commonly found in crystal structures of Fab fragments [33]. The final electron density is of good quality with well-defined polypeptide chains. Nevertheless, residues 127–132 are not well defined in the electron density indicating disorder in this region. These residues form a loop in the first constant region of the heavy chain (C_H1_), which is also not well defined in crystal structures of other Fab fragments [34]. All 7C8 residues were numbered according to the Chothia numbering scheme that compensates for length variability in the CDRs and generates a consistent numbering of the structurally conserved Fab framework region [35].

**Table 1:** Data collection and refinement statistics.

Statistics of diffraction data	
Wavelength (Å)	0.97 Å
Resolution (Å)	44.6–2.7 (2.85–2.7)
Space group	P3_2_21
Unit-cell parameters (Å)	*a* = *b* = 100.1, *c* = 196.8
*V*_M_ (Å^3^ Da^-1^)	3.03
Solvent content (%)	59
No. of molecules in ASU	2
No. of observed reflections	245681 (31362)^a^
No. of unique reflections	32045 (4588)
Redundancy	7.7 (6.8)
Completeness (%)	99.9 (99.9)
*R_merge_* b (%)	7.0 (62.1)
〈I/σ(I)〉	21.1 (2.3)

Statistics of refinement and structure model	
*R*_cryst_ c (%)	22.6
*R*_free_ d (%)	27.3
Number of protein atoms	6677
Number of water molecules	78
Number of glycerol molecules	1
RMSD from ideal geometry	
bond lengths (Å)	0.007
bond angles (°)	1.065
Average B factor (Å^2^)^e^	
Residues	88
Water	29
Glycerol	93
Ramachandran plot (%)	
Most favored regions	90
Allowed regions	9.6
Generously allowed regions	0.1
Disallowed regions	0.3

a Number in parentheses indicate the outer-resolution shell.

b *R*_merge_  =  ∑*_hkl_* ∑*_i_* |*I_i_* (*hkl*) - 〈*I* (*hkl*) 〉|/∑*_hkl_* ∑*_i_ I_i_* (*hkl*), where *I_i_*(*hkl*) is the *i*th observation of reflection *hkl* and 〈*I* (*hkl*) 〉 is the weighted average intensity for all observations *i* of reflection hkl.

c *R*_cryst_  =  Σ_hkl_  =  ∑_hkl_|*F*_obs_ − *F*_calc_|/Σ_hkl_ |*F*_obs_|.

d *R*_free_ is the same as *R*_cryst_ except for 5% of the data excluded from the refinement.

e Sum of the TLS and Residual B-factor contributions.

### Overall structure of the 7C8 Fab fragment

**Figure 1:**
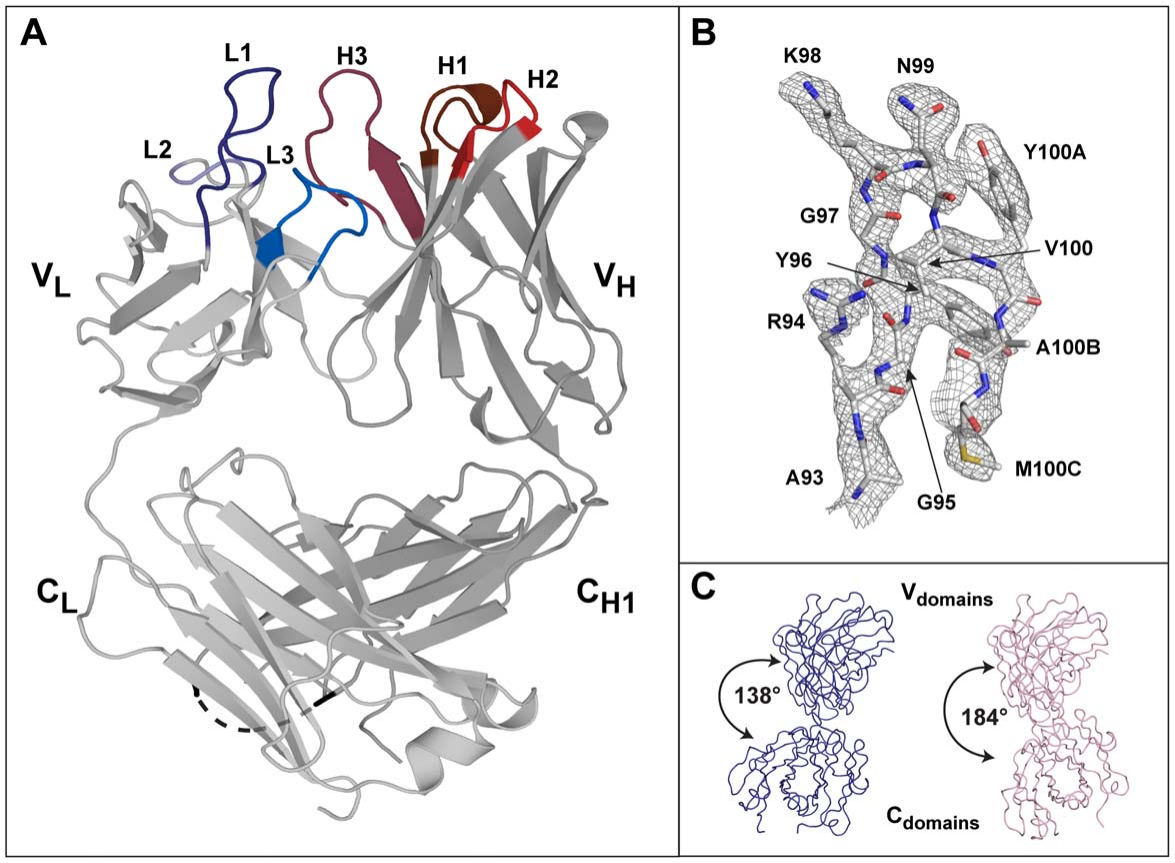
Overall three-dimensional structure of the HIV-2 neutralizing Fab fragment 7C8. A) Overall view of the crystal structure of the 7C8 Fab fragment. The light and heavy chains are displayed in light and dark grey, respectively. The six CDR loops are in dark blue (CDRL1), light blue (CDRL2), blue (CDRL3), brown (CDRH1), red (CDRH2) and pink (CDRH3). A black dashed line indicates the disordered loop within the first constant region of the C_H1_ domain. B) Stick representation of the stretch of residues 93 to 100C corresponding to the unusually long third complementary determining region of the heavy chain (CDRH3) with the electron density carved around the model at 1.2 σ. **C**) Ribbon representation of the two monomers found in the assymmetric unit indicating the large variation in elbow angle.

The overall structure of the 7C8 Fab fragment comprises the four immunoglobulin domains V_L_, V_H_, C_L_ and C_H1_ that form the variable (V) and constant (C) domains of the light (_L_) and heavy chains (_H_) (Figure 1A). Two pairs of disulfide bridges are formed between cysteine residues 23-88, and 134–194 in the light chain as well as between cysteine residues 22–92 and 140–195 in the heavy chain. The binding site of the 7C8 Fab is formed by the six solvent-exposed CDR loops CDRH1, H2, H3, L1, L2 and L3, which project from the β-strands of the V_H_ and V_L_ domains (Figure 1A). All residues that form the six CDRs, including the prominent side-chains of the CDRH3 residues K98 and N99, have well-defined electron density (Figure 1B).

The elbow angle, which is commonly used to describe the relative orientation of the variable *versus* the constant domains of each Fab fragment, differed significantly between the two 7C8 molecules present in the asymmetric unit with 138° and 184° for 7C8 monomer 1 (chains A and B) and monomer 2 (chains H and L), respectively (Figure 1C). Nevertheless, this variation lies within the range commonly found for Fab fragments with a kappa light chain [36]. The flexibility of the elbow likely reflects an adaptation to circumvent potential sterical hindrance and to allow bivalent binding for large immunoglobulins [36]. Packing of the two 7C8 monomers in the asymmetric unit results in a limited number of crystal contacts including interactions that possibly favor the observed differences in elbow angle and the stabilization of the CDRs (data not shown). However, it should be noted that the conformation of all the residues comprising the six CDR loops is identical when comparing the 7C8 monomers 1 and 2 in the asymmetric unit.

### The deep and narrow hydrophobic antigen-binding site of 7C8, bordered by the unusually long CDRH3 domain, is well fitted for binding of an elongated epitope

**Figure 2:**
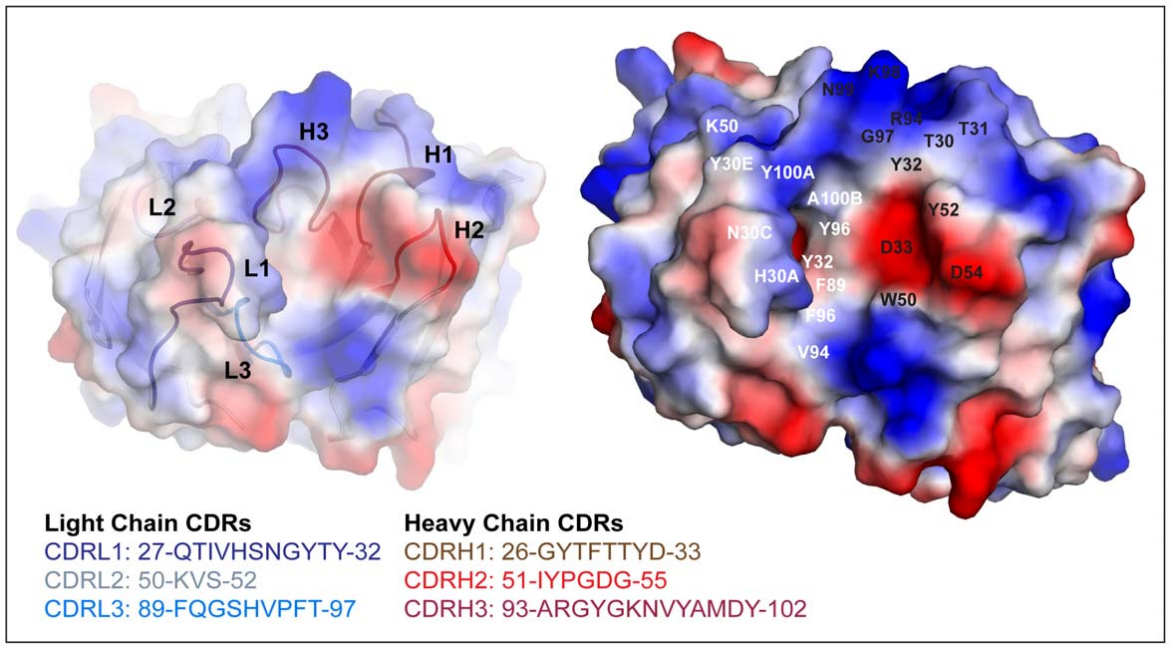
The deep and narrow antigen-binding site of 7C8, well fitted for elongated epitopes, is highly hydrophobic. The surface of the antigen-binding site of 7C8, displayed from the perspective of a bound antigen, is colored according to its electrostatic surface potential. To the left, the six CDR loops that form the antigen-binding site are colored according to the sequence-panel. To the right, the relative arrangement and the conformation of the CDRs result in the formation of a deep and narrow highly hydrophobic cleft surrounded by the prominent CDRL1, CDRH1 and CDRH3 loops. Each side of the deep antigen-binding cleft is positively (in blue) and negatively (in red) charged, respectively, while the middle section is highly hydrophobic. A selection of light and heavy chain residues that form the antigen-binding site are indicated in white and black, respectively.

Five of the six CDRs in 7C8 (H1, H2, L1, L2 and L3) display conformations that correspond to canonical classes 1, 2, 4, 1 and 1, respectively (Figure 2) [37]. The CDRH3 loop of 7C8 comprises 13 amino acid residues and is therefore elongated with respect to the average length (8–9 residues) of mural IgG molecules [31], [32], [38]. It features a kink, often found in Fab fragments of antibodies, that results from the formation of hydrogen bond interactions between the pairs of residues R94-D101 and M100C-W103 [37], [39] (data not shown). These canonical classes are most commonly found in so-called peptide-binding antibodies and, combined with shorter CDRH3 loops, are usually attributed to the presence of a deep cleft within the antigen-binding site in contrast to flatter antigen binding interfaces found in so-called protein-binding antibodies [38], [40]. An example of an antibody belonging to this family is K42-41L, that shares high sequence similarity with the heavy chain of 7C8 (80% identity), and which CDRs display similar conformations to those found in 7C8 [41]. The Fab fragment of K42-41L, that binds a loop on rhodopsin, was crystallized with a peptide mimic of the epitope that fits snuggly in the peptide-binding cleft of the antibody. A similar pattern with a conserved peptide epitope, derived from the V3-region of gp120, running through an antigen binding cleft bordered by an elongated CDRL1 and a much shorter CDRH3 has also been described in the crystal structure of the murine anti HIV-1 antibodies 83-1 and 50-1 [42], [43].

Nevertheless, and as also previously observed in the murine anti HIV-1 V3 antibody 58.2 [44], the CDRH3 loop of 7C8, localized at one end of its deep, narrow and highly hydrophobic cleft (Figure 2) is unusually long. Although the rationale underlying the apparent need for such an elongated domain is not fully understood, one possible explanation could be that the unusual length of CDRH3 allows for extensive contacts with the V3-derived peptide as nicely exemplified in the crystal structure of the human anti HIV-1 V3 antibodies 447-52D, 3074 and F425 [20], [45], [46]. A second non-excluding possibility could be that the unusual length of this loop allows the antibody to overcome potential sterical hindrance and reach its epitope with higher affinity. In conclusion, the extended conformation of the CDRL1 and CDRH3 loops in the antigen-binding site of 7C8 may allow extensive interactions with the V3 region of gp125 and possibly to overcome sterical hindrance.

Qualitative analysis of the electrostatic surface potential indicates that the two sides of the deep antigen-binding cleft are positively and negatively charged, respectively (Figure 2). The deepest middle section, formed by residues Y30E, Y32, W50, F89, V94, F96, Y96, G97, Y100A and A100B, is hydrophobic. Most of the positively charged side of the cleft is composed by residues K50, R94 and K98, localized on CDRL2 and CDRH3, while the negatively charged side of the antigen-binding site is mainly formed by the aspartate residues D33 and D54, localized on CDRH1 and CDRH2, respectively (Figure 2). Interestingly, the six CDRs feature a high number of tyrosine, histidine and phenylalanine residues. The relative over-representation of tyrosine residues seems to be a common feature for protein-specific IgGs [38] and, similarly to what has been previously described, such aromatic side chains could potentially play an important function in docking and affinity of epitopes through the creation of multiple weak interactions with the target [47], [48].

In conclusion, the crystal structure of the Fab fragment of 7C8 reveals a narrow highly hydrophobic antigen-binding cleft, surrounded by charged and aromatic residues. Although five of the CDR loops feature canonical conformations, it should be noted that the antigen binding-cleft of 7C8 also features an unusually elongated CDRH3.

### Molecular modeling indicates that the main epitope FHSQ of 7C8 is exposed and localized centrally on the stem of the V3 domain of gp125

A molecular model of the gp125 monomer was generated using as template a crystal structure of gp120 that includes the V3 region [18]. Additionally, a putative trimeric model of gp125 was created using the previously published molecular model of the gp120 trimer [49] in order to study binding of 7C8 in its functional context.

**Figure 3:**
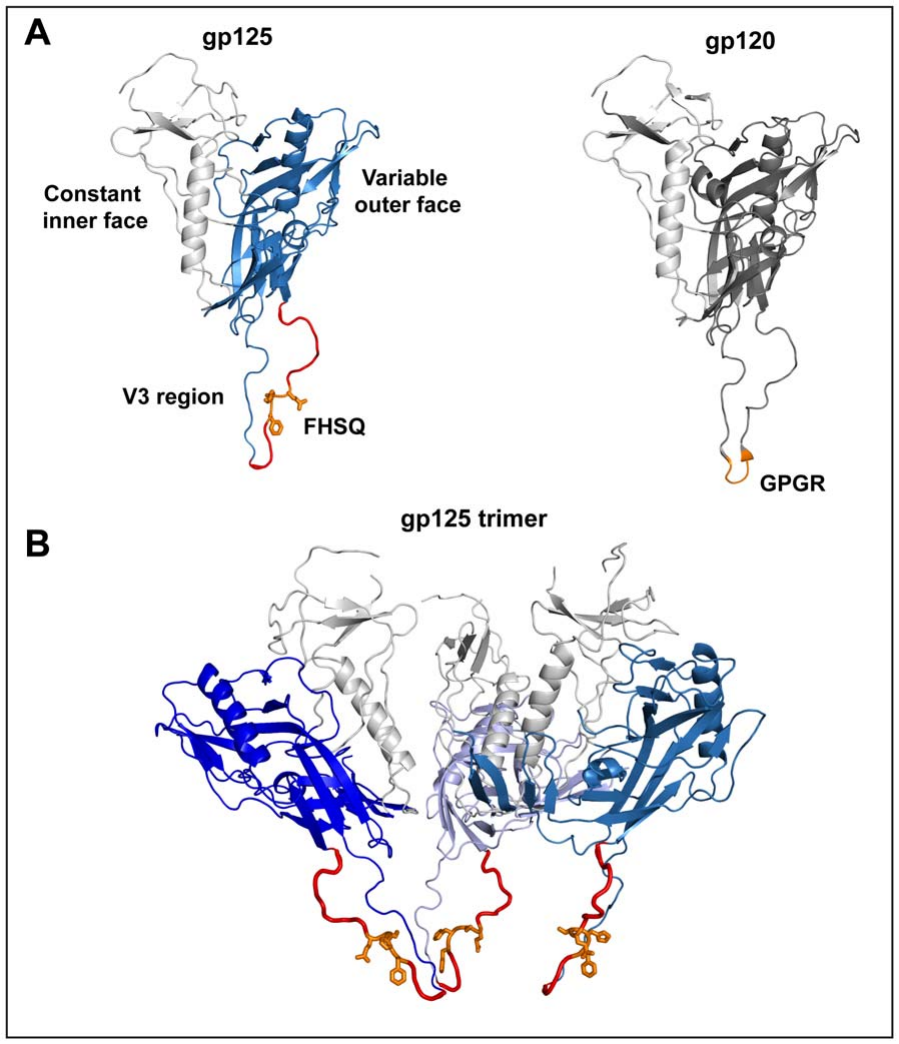
The epitope FHSQ is localized centrally on the V3-region of gp125. **A**) The molecular model of gp125 is very similar to its HIV-1 counterpart gp120. The conserved inner face and the more variable outer face of gp125, which includes the V3 region, are highlighted in grey and blue, respectively. The 15-mer gp125 V3-peptide used to raise 7C8 is displayed in red. The localization and solvent exposure of the epitope GPGR on gp120 and the epitope FHSQ on gp125 are highlighted in orange. **B**) A putative trimeric model of gp125 displayed with the V3 region oriented towards the target cell indicates the accessibility and solvent exposure of the FHSQ epitope.

The antibody 7C8 was originally isolated from mice immunized with a 15-mer peptide, derived from the V3 region of gp125, and corresponding to residues 326–341 (SGRRFHSQKIINKKPR) [27]. It has also been previously demonstrated that the specificity of 7C8 is centered on the stretch of V3-residues 330–333 FHSQ [50]. The molecular model of gp125 indicated that the conserved immunodominant epitope FHSQ is localized in the middle part of the V3 region (Figure 3A), while the trimeric model further suggested that all residues forming the FHSQ epitope protrude towards the solvent, readily available for binding (Figure 3B). Interestingly, the localization of FHSQ residues does therefore not correspond to that of the seemingly analogous conserved and immunogenic HIV-1 epitope GPGR, which constitutes the tip of the V3 region (Figure 3A) [18] and the two HIV epitopes do not align when comparing the amino acid sequences of gp120 and gp125 (data not shown). It should be noted that detailed structural information about the conformation of the V3 region is only available for the CD4-bound form of gp120 [18]. The V3 region could be less exposed prior to CD4 binding and might display a different orientation in the trimer [5], [7], [10], [51]. On the other hand, it has also been previously demonstrated that 7C8 binding to trimeric gp125 was not enhanced by the removal of both the V1 and V2 loops [50].

### A molecular model of 7C8 in complex with trimeric gp125 suggests sterical hindrance as a potential mechanism, underlying its efficient capacity to neutralize HIV-2

The program HADDOCK [52], [53] was used to model possible docking solutions between the crystal structure of the 7C8 Fab fragment and the molecular model of monomeric gp125. It should be noted that in order to limit bias in the docking procedure, the entire sequence that was originally used to raise the antibodies in mice (residues 326–341 SGRRFHSQKIINKKPR) was used as a target for the docking of 7C8 on the molecular model of gp125. Relevant amino-acid residues (with more than 40% accessible surface area) in L1, L2, L3, H1, H2 and H3 were chosen as docking partners on the crystal structure of 7C8. Flexibility of the side chains and backbone of the V3 domain and the 7C8 CDRs was allowed during the simulated annealing that formed the basis of the final rounds of refinement [52], [53]. This is likely to reflect the expected flexibility of the V3 domain as well as the limited conformational changes commonly observed in most CDRs upon ligand binding [18], [54], [55].

**Figure 4:**
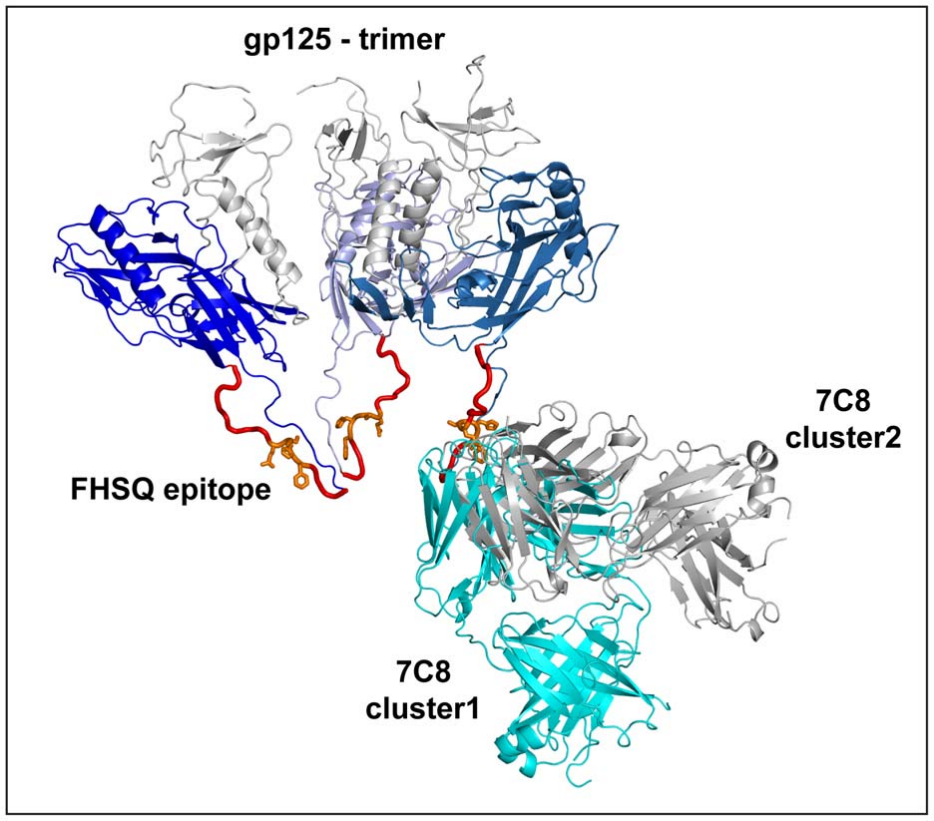
Docking simulations indicate that the antigen-binding site of 7C8 binds primordially to the FHSQ epitope on gp125. Representation of the two docking solutions of the crystal structure of 7C8 in complex with the putative gp125 trimer. The two solutions, displayed in cyan and grey respectively, interact mainly with the solvent-exposed FHSQ epitope. The interactions proposed by the two solutions also suggest a mechanism for neutralization since the size of the bound molecules would not allow for subsequent engagement of the gp125 trimer to the co-receptor on target cells.

Analysis of the resulting docking clusters provided two main docking solutions. Although the relative position of 7C8 towards gp125 was significantly different in the two docking solutions, their antigen-binding sites bound to the same part of the V3 domain, focusing on the epitope FHSQ (Figure 4). The two solutions could be superposed onto a trimeric model of g125 without sterical clashes (Figure 4). It should also be noted that no clashes were observed when the superposition was performed following the addition of basic glycan-structures to predicted glycosylation sides on the trimeric gp125 model (data not shown). However, due to the uncertainties and limitations of scoring and validation [56], [57] and the large differences between the two main solutions, we will not provide a detailed analysis of how the 7C8 Fab fragments are docked on gp125.

Importantly, besides enforcing the importance of the FHSQ epitope for recognition by 7C8, the two models provide a potential mechanism for neutralization by 7C8 as the size of bound full IgG or Fab fragments would not allow for subsequent engagement of the gp125 trimer with the co-receptor on the target cell.

### Concluding remarks

The three-dimensional crystal structure of the Fab fragment of the mouse monoclonal antibody 7C8, specific for the third hypervariable region (V3) of gp125, reveals a deep and narrow highly hydrophobic antigen-binding cleft with architecture appropriate for a linear epitope. The CDRH2, CDRH3 and the CDRL1 domains border this cleft. Molecular docking analysis provides two main clusters of solutions that both indicate the high specificity of 7C8 to the main epitope composed by the stretch of residues FHSQ, which are localized centrally on the V3 region of gp125.

## Materials and Methods

### Production and purification of 7C8 Fab fragments

The production, purification and crystallization of the 7C8 Fab fragments have been described previously [58]. Briefly, 7C8 was expressed from hybridomas and Fab fragments were prepared through limited digestion with papain (Sigma). The Fab fragments were subsequently purified using size-exclusion chromatography and concentrated to 10 mg/ml.

### Crystallization of 7C8 and data collection

Crystals of 7C8 Fab fragments were obtained in 100 mM Tris-Cl pH 8.5, 50 mM ammonium sulphate, 25% (*w/v*) PEG 8000 and 2.5% (*v/v*) PEG 400 at 20°C by hanging-drop vapor diffusion [58]. The 7C8 crystals diffracted to a resolution of 2.7 Å and belong to space group *P3_2_21*, with unit-cell parameters *a* = *b* = 100.1 Å, *c* = 196.8 Å. The asymmetric unit contains two 7C8 molecules with a molecular mass of 47.1 kDa, giving a solvent content of 59%. Space group and unit cell parameters were determined using the auto-indexing option of MOSFLM [59]. Scaling and reduction of the data were performed using SCALA from the CCP4 suite of programs [60]. Five percent of the reflections were set aside for validation. Data-collection statistics are summarized in Table 1.

### Structure solution and refinement

The three-dimensional structure of the Fab fragment of 7C8 was solved by molecular replacement using the programs PHASER [61] and MOLREP [62]. The atomic coordinates of the light and heavy domains of the recombinant anti-testosterone (3-C(4)F(5)) Fab fragment (PDB ID 1I9J; [63]), including all side chains, were used as search models. Solutions for all domains of the two Fab molecules in the asymmetric unit could be determined except for one light chain. Using the solution from PHASER as a fixed model in MOLREP, the solution for the lacking light domain was also determined. Rigid body refinement in REFMAC5 [62] was followed by a round of simulated annealing using the program CNS [64]. Further restrained refinement was carried out using REFMAC5 applying TLS parameters in the final rounds of refinement [65], [66]. Model building was performed using O [67] and COOT [68]. Residues 127 and 132 are part of a disordered loop and were modeled with occupancy of 0.7. The geometry of the final model was analysed using PROCHECK [69]. The final model consists of 869 residues. A total of 78 water molecules were added to well-defined peaks (2.0s and greater in F_o_-F_c_ electron density maps) found between 2 and 4 Å from oxygen or nitrogen atoms. The final refinement statistics are listed in Table 1. The 7C8 residues were numbered according to Chothia *et al.*, [35] using the Abnum numbering server (http://acrmwww.biochem.ucl.ac.uk/abs/abnum/; [70]). The elbow angle was calculated using the program RBOW (http://proteinmodel.org/AS2TS/RBOW/index.html; [71]). Qualitative vacuum electrostatic surface potentials were evaluated through calculation of locally averaged surface charges using the protein contact potential visualization as implemented in Pymol (PyMOL Molecular Graphics System, Version 1.2r1, Schrödinger, LLC). All figures were prepared using Pymol.

### Molecular modeling

A molecular model of the HIV-2 gp125 monomer (subtype SBL6669_isy_; GeneBank ID J04498) was generated using the SWISS-MODEL protein modelling server [72] based on the published crystal structure of the V3-containing gp120 molecule (PDB ID 2B4C; [18]). The two sequences could be well aligned despite limited sequence identity (36%). Insertions and deletions in the sequence of gp125 when compared to gp120 were almost exclusively confined to loops or turns in the variable outer domain of the molecule. The molecular model of the gp125 trimer was created by superposing the monomer on the coordinates of a previously published model of the HIV-1-associated gp120 trimer [49], generously provided by Prof. Peter Kwong (Vaccine Research Center, NIH, Bethesda, MD, USA). Despite small differences in geometry and relative disposition of the V1/V2 loops and the V3 region, the topology of the trimer model is in good overall agreement with the three-dimensional structures derived from cryo-electron tomography [5], [7], [51].

### Docking simulations

Docking was performed using the program HADDOCK [52], [53], with the default settings of the program. Initial rigid body docking was performed from 1000 starting configurations with the two proteins randomly oriented. The 200 docking solutions with the lowest overall energy (score) were selected for further development. The second stage of docking comprised simulated annealing refinements that allowed increased flexibility of side chain and backbone torsion angles. Water molecules were added in the final step of docking refinement in order to optimize surface interactions. The obtained molecular models were scored again and grouped thereafter into clusters using an algorithm described by Daura *et al.*, [73], based on a 7.5 Å cut-off. The quality of the clustering was checked by visual inspection of the two-dimensional projection of the clusters according to the algorithm suggested by Levitt [74] and as implemented in the CHARMM program [75]. Here each of the N structures is associated with a point (Pi, Qi), and the parameters (Pi,Qi)^i = 1,N^ are optimized to minimize the function 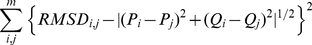
(1)


where RMSD_i,j_ is the root mean square deviation between structures i and j, and the sum is over all m pairs of structures. The clusters were ranked by their size and scores, and the two most prominent solutions were chosen for further analysis.

### Accession Numbers

The atomic coordinates and structure factors of the HIV-2-neutralizing Fab fragment (PDB ID 3NZ8) have been deposited in the Protein Data Bank (www.pdb.org; [76]). The coordinates of all models described in this study will be provided upon request.
